# Electricity as a Therapeutical Means in the Treatment of Lateral Spinal Curvature

**Published:** 1892-09

**Authors:** Hugh Hagan

**Affiliations:** Atlanta, Ga.


### ELECTRICITY AS A THERAPEUTIC MEANS IN THE TREATMENT OF LATERAL SPINAL CURVATURE.

BY HUGH HAGAN, M. D.,
ATLANTA, GA.

It is not my purpose to discuss at length the subject of spinal lateral curvature, but to suggest the possible advantages of electrical stimulation as a therapeutic means in the treatment of spinal curvature.

This condition is among the very frequent deformities of the present age, and to find out the best and quickest means to remedy the deformity should be our aim. It behooves us to try all means that promise any favorable result, and not to condemn or refuse to try a suggestion until the suggestion proves futile.

After a thorough study of the methods taught and practiced, I have failed to find mention of electricity applied to this especial condition. Authors good and poor of recent times all quite agree as to the etiology, pathology and treatment of this condition. My remarks will be confined to the treatment only, as the etiology and pathology are accepted conditions, and possible progress alone remains to treatment.

Without exception, authorities recommend general tonics, physical exercise, particularly for the weakened muscles, braces, plaster corsets, prolonged dorsal decubitus, sleeping on the side, with some such object as a pillow interposed, the slanting stool, shorter hours at school, no strain on affected side, etc., while the stimulating and developing powers of electricity are quite ignored. Even Dr. Lewis A. Sayre, of New York, our most eminent orthopedic surgeon, always practical and original—though practicing all other methods—fails to mention the good effects and legitimate demands of faradic electricity. Though great and glorious results have resulted from the use of the plaster of Paris corset, gymnastic exercises and tonics, yet I think had electricity been tried the results would have been achieved in much less time and with far less discomfort. I have suggested the use of electricity to one or two of my friends as a means to be used in this condition, but they failed to apply it, and it was left to me so to do. I doubted not the correctness of my idea, for the general use of the fluid in cases of atrophies, as atony of other muscles, was of daily occurrence and obvious advantage. I would have applied it sooner, but as my special work in medicine, the diseases of the mind and nervous system, kept me from seeing as many cases as I possibly otherwise would have seen, I did not have an opportunity until four months ago.

The patient on whom it was tried was a young girl, Miss M. L., age 16, dark brunette, tall, very thin, with extremely small chest, weak heart action, and presenting a picture of chlorosis quite marked. She was brought to me on account of the frequency of her headaches, restlessness, listlessness, insomnia, irregular menstruation and general nervousness. She was obstinately constipated, and, as her mother (step-mother) expressed it, "had a crooked back."

The deformity was caused by a very marked lateral curvature. The curvature was of a right dorsal type, with compensating cervical and lumbar curves. The right shoulder was much lower than the left, the inferior angle of the right scapula one inch lower than the left, the shoulder very prominent, the dorsal spinal process 1¼ inches out from a perpendicular, dropped from the occipital protuberance, with the cervical and lumbar curvatures almost as decided.

I began her treatment with aloin, strychnine and belladonna pills, train and cod liver oil, shorter hours at school, exercises to develop all weakened muscles, and faradization. The electrical treatment consisted of general and special stimulation of each and every muscle in neck and back. The current was strong enough to produce a decided contraction, and the sittings about twenty minutes daily.

The improvement was noticeable in six weeks, and at the end of four months the patient was discharged, she having gained in weight, strength and general good health, and the spinal deformity reduced to a minimum. The inferior angles of the scapulæ were on a line, the cervical and lumbar curves connected and, save a permanent deviation hardly to be measured in the upper dorsal region, the spinal processes in a line. The erectores spinæ were well redeveloped and equally prominent on both sides.

Now it may be said that the exercise, tonics, short hours at school, good hygiene and dietetics did their share of the work, which I am ready and willing to acknowledge, while the faradization was only placeboic, yet I claim that without plaster corset, braces or other like appliances, a well marked case of spinal curvature was cured in four months, while twelve, eighteen and more months are occupied with the old methods.

I hope this suggestion as to the further advantages of electricity as applied, may induce others to try it, and I doubt not but all satisfaction will be received, for I see no reason why an erector spinæ, an intercostal, as well as any other muscle, weakened by disease, disuse, overstrain, etc., may not be permanently benefited by electrical stimulation.

211 *Peachtree Street, Atlanta, Ga.*

